# A switch from horizontal compression to vertical extension in the Vrancea slab explained by the volume reduction of serpentine dehydration

**DOI:** 10.1038/s41598-022-26260-5

**Published:** 2022-12-24

**Authors:** Andreea Craiu, Thomas P. Ferrand, Elena F. Manea, Johannes C. Vrijmoed, Alexandru Mărmureanu

**Affiliations:** 1 National Institute for Earth Physics, Calugareni, 12, Măgurele, Ilfov, Romania; 2 Institut für Geologische Wissenschaften, Freie Universität Berlin, Malteserstraße 74-100, 12249 Berlin, Germany; 3 Institut des Sciences de la Terre d’Orléans, UMR-7327, Université d’Orléans – CNRS, 1A Rue de la Ferollerie, 45100 Orléans, France; 4 GNS Science, PO Box 30-368, Lower Hutt, New Zealand

**Keywords:** Planetary science, Solid Earth sciences, Geodynamics, Geophysics, Seismology, Tectonics

## Abstract

The Vrancea slab, Romania, is a subducted remnant of the Tethyan lithosphere characterized by a significant intermediate-depth seismicity (60–170 km). A recent study showed a correlation between this seismicity and major dehydration reactions, involving serpentine minerals up to 130 km depth, and high-pressure hydrated talc deeper. Here we investigate the potential link between the triggering mechanisms and the retrieved focal mechanisms of 940 earthquakes, which allows interpreting the depth distribution of the stress field. We observe a switch from horizontal compression to vertical extension between 100 and 130 km depth, where the Clapeyron slope of serpentine dehydration is negative. The negative volume change within dehydrating serpentinized faults, expected mostly sub-horizontal in the verticalized slab, could well explain the vertical extension recorded by the intermediate-depth seismicity. This apparent slab pull is accompanied with a rotation of the main compressive stress, which could favour slab detachments in active subduction zones.

## Introduction

**Figure 1:**
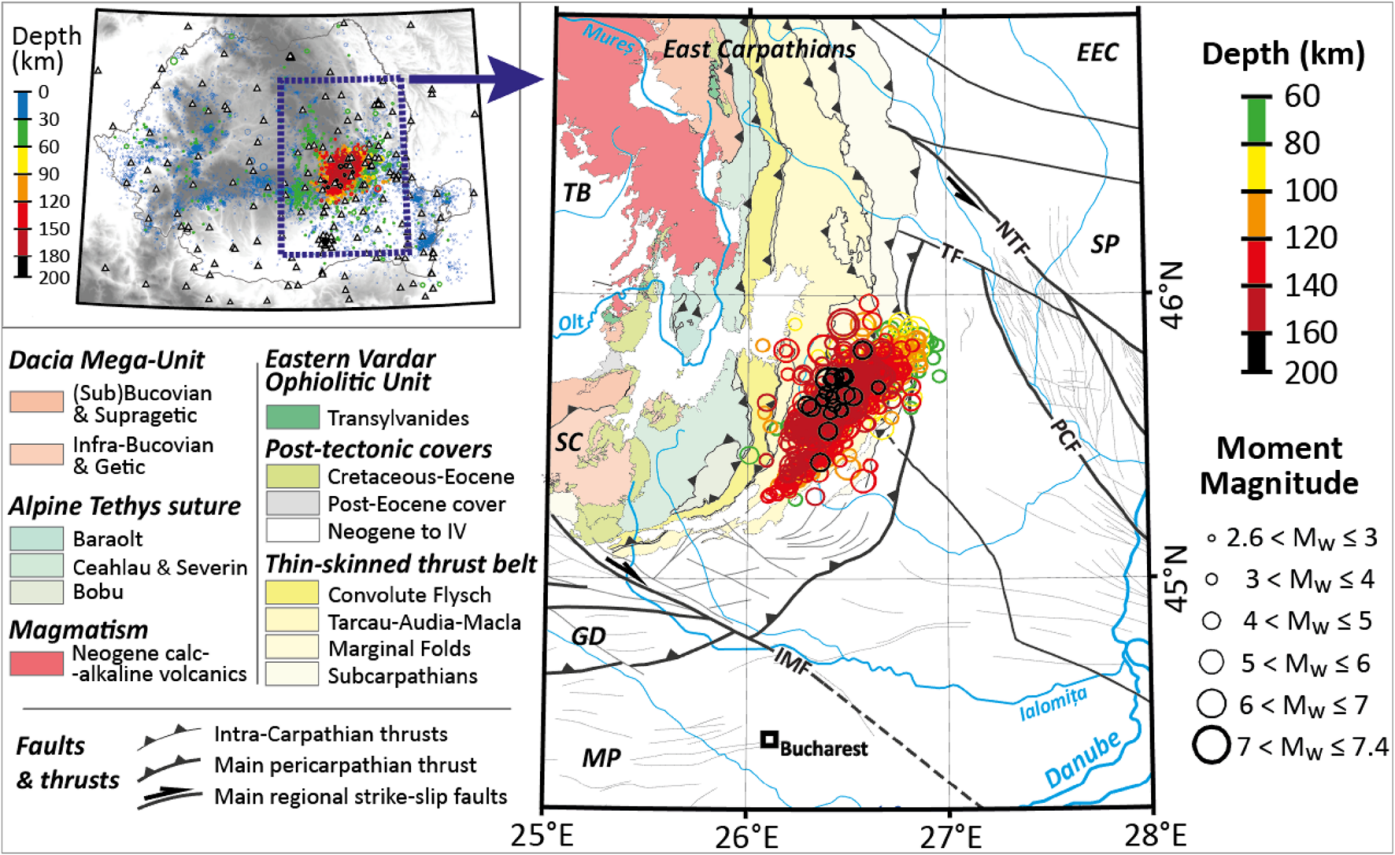
Synthetic map showing main tectonic units and intermediate-depth seismicity beneath Vrancea (modified after^9^). The reduced dataset of 940 events for the 1929–2020 period is presented in Table S1. Hypocentral depths are indicated with the colour scale. The study area is presented with the purple contour on the mini-map. Map construction: tectonic features from^4^, epicentral locations from the BIGSEES and ROMPLUS catalogues. EEC: East European Craton; GD: Getic Depression; IMF: Intra-Moesian Fault; MP: Moesian Platform; NTF: North Trotuș Fault; PCF: Peceneaga-Camena Fault; SC: South Carpathians; SP: Scythian Platform; TB: Transylvania Basin; TF: Trotuș Fault.

The Vrancea seismic zone is located in the bend region of the South-Eastern Carpathians, in Romania. It is a unique area with both shallow and deep seismic activity (Fig. 1), known as one of the most active intermediate-depth seismic areas in Europe^1–3^. Shallow seismicity (< 30 km depth) affects crustal materials, while intermediate-depth seismicity (from 30 to 170 km) has triggered intense debates (e.g.^4–8^). According to most recent findings, the latter would correspond to ruptures occurring within a remnant slab of subducted lithospheric mantle, whose location is explained by slab retreat and limited continental delamination during the late Neogene^9^. Moderate crustal seismicity is recorded all over the Carpathian region, but the far more intense and persistent activity occurs in a small subcrustal seismogenic volume beneath the SE bend of the Carpathian Arc. The Carpathians collision peaked during the Miocene until 8–9 Ma, when the subducted slab was located below the Transylvanian Basin, and was followed by large-scale differential motions within the orogen and its foreland (limited shortening), still active in the SE Carpathians and thought to be related to the deep evolution of the Vrancea seismic body (e.g.^4,8^).

The Vrancea seismic body shows a 70-km SW-NE alignment with a lateral extent of about 30 km. Its vertical extent is ≈ 110 km, between 60 and 170 km^10^, with some events occurring up to 200 km depth. Over the last century, four earthquakes with moment magnitudes *M*_W_ > 7 occurred in this region^11–13^ and significant damage was reported in neighbouring countries (e.g.^1^). This seismic activity is generated within a relic slab located in a nearly vertical position in the Earth’s mantle due to limited delamination of the Carpathian lithosphere. Several geodynamic models have been proposed to explain its origin, ranging from slab retreat and roll-back to continental delamination, slab detachment or a combination of the latter. Some studies argue for a gravitational instability^5,13^. The debate on the nature and origin of the Vrancea seismic body has recently been detailed in the form of a transdisciplinary review^9^.

The overall distributions of stresses and strain rates within the Vrancea slab are the result of external forces, which may include pulling forces, convergence-induced horizontal compression and gravitational instabilities. The stress field is explained by Newton's laws and by the density distribution in the studied volume. Numerical modelling reported abnormally high strain rates in the seismic volume (> 20 mm yr^-1^)^5^, but these results rely on only 31 events and uncertain parameters and do not consider mineralogy and transformations. Stress accumulations within subducted slabs build up due to rheological heterogeneities (e.g.^14–16^), for instance at the scale of peridotite blocks elastically loaded in between serpentinized faults^9,17^. Significant Tertiary deformations in Vrancea as well as intra-Carpathian block rotations resulted from actual slab pull forces during slab verticalization until the mid-Miocene, along with lateral tectonic forces (Adria push), and both types of forces appear to have considerably decreased at present^6^. Vertical stresses ≤ 90 MPa are expected^6^; for comparison, bending/unbending during active subduction can build stresses > 1000 MPa, as further supported by most recent numerical modelling^16^. Then, for a given elastic stain (stored elastic energy), the nucleation of seismic ruptures is due to critical stress distortions relying on local processes, i.e. mineral destabilizations (e.g.^14,17,18^).

A link with mineral transformations has recently been shown, strongly suggesting that the Vrancea deep seismicity is due to transformation-driven stress transfers upon dehydration reactions during slow heating at a given pressure^9^. According to the latter, we favour the oceanic nature of this slab. In any case, the relatively high deviatoric stress within this volume is partly controlled by the density distribution of the slab and surrounding materials^19,20^, from which the elastic energy released during earthquakes originates. The triggering mechanism of this seismicity requires local stress amplifications, interpreted as either due to an increase in the stress applied to this volume^19^ or due to transformation-driven stress transfers upon dehydration reactions during slow heating at constant pressure^9^.

The unique slab geometry at the Carpathians corner causes stress localization. The bent nature of the Carpathian mountain belt during the recent collision necessarily induced a regional stress amplification, further followed by additional deformations during slab verticalization and associated delamination. Slab bending, unbending and subsequent stress release have been considered as the main cause for large mantle earthquakes in the region^21^, but the recent discovery of the correlation between seismicity and dehydration reactions highlights the need for further investigations^9^. Knowing about the stress state and orientation of the stress field within the slab could help grasp the mechanisms involved. Therefore, a better understanding of the link, if any, between triggering mechanisms^9^ and focal mechanisms^22,23^ of the seismic ruptures is needed.

The dehydration of serpentine minerals and other hydrous phases demonstrably triggers seismicity at intermediate depths within actively subducting slabs^14,17,18,24,25^. Experimental findings show that dehydration-induced ruptures in slightly serpentinized peridotites do not require fluid overpressure^14^, in contrast with the prediction of the “dehydration embrittlement” model. These experimental results notably allow us to understand seismological data from the lower Wadati-Benioff plane of seismicity^26^. Nonetheless, because the correlation with stability limits of serpentine minerals does not account for the entire seismological datasets, the “dehydration-driven stress transfer” model was generalized to any minor/local mineral transformation. Various dehydration reactions or other fast transformations of local mineral clusters are expected to participate in this seismicity^9,17^. Mechanical instabilities can be triggered by transformation-driven stress transfers as soon as the unstable mineral reaches the limits of its stability field^18,27–29^.

Contrary to a persistent belief, the lower Wadati-Benioff plane of seismicity in actively subducting slabs does not correspond to the dehydration “isotherm” of serpentine, but rather to the depth limit of serpentinization within the incoming oceanic lithosphere before subduction^14,17,18^. The latter can be locally hydrated through different kinds of faults, including bending faults (e.g.^30,31^), spreading faults (e.g.^32^), transform faults and fracture zones (e.g.^33^). The distribution of serpentinized faults is responsible for the distribution of seismic events at depth, due to rheological contrasts^16^ eventually amplified by mineral destabilizations such as either dehydration reactions^26^, hydration reactions^34,35^ or indirect consequences of the latter^36^.

Bending faults are ubiquitous at subduction zones as they are directly related to lithospheric bending prior to subduction. These bending faults can extend down to the brittle-ductile transition, 20–40 km below the seafloor^30,31^ and were identified worldwide offshore subduction trenches using various geophysical methods^31,37,38^. Water infiltration and consecutive hydration reactions occur along these faults^30–33^. Because serpentine minerals are the main hydrous phases constituting these hydrated faults, they are named “serpentinized faults”, which does not necessarily mean that other phases from the talc or chlorite families, for example, cannot be included^17^. Although pervasive serpentinization is inhibited at high pressure (> 1 GPa), faulting and fault reworking at trenches favour transient H_2_O percolation through the fault network, in which the serpentinization front can self-propagate^39,40^ and possibly cause additional stress transfers and strain localization events^34,35^. Deep percolation of transient fluids upon serpentine dehydration is supported by both field geology^41,42^ and seismological observations^43^.

The understanding of intermediate-depth seismicity has benefited from field observations (e.g.^15,34,39–41,44^, laboratory experiments (e.g.^14,34,45,46^) and transdisciplinary comparisons (e.g.^26,47^). Both negative and positive volume changes during mineral reactions are associated with earthquake triggering, either in the laboratory^14^, in actively subducting slabs ^24,25^ or in the Vrancea slab ^9^. At depths above ≈ 60 km (≥ 2 GPa), the volume change of antigorite dehydration becomes negative. In this pressure range, several dehydration reactions theoretically result in pore pressure reduction, thus models based on fluid overpressure cannot apply ^14,48^, which makes hydrofracking-like events (“dehydration embrittlement”) definitely impossible. This urged scientists to think about alternative mechanisms to interpret shear failures at such high pressures^14,45,49^.

Whatever the trigger, dynamic ruptures are only possible if a sufficient amount of elastic strain is stored in the bulk rock. Numerous field studies report significant stress values recorded by pseudotachylytes (e.g.^44,50^). Recent numerical modelling shows that the stress can demonstrably build up due to scattered rheological contrasts within the sinking slab during unbending^16^. Yet, a recent study highlights that dehydration-induced seismicity also occurs within the Vrancea slab, although it is not subducting anymore^9^, showing that residual stresses within locked subducted remnants are seismically released as a result of local stress perturbations^9^. Dehydration reactions are not intrinsically seismic^14,34,46^, but it occurs fast enough to trigger stress transfers into the surrounding peridotites^14,27–29^. The sudden grain size reduction is a key parameter controlling the stress transfer and rupture nucleation (e.g.^51^), required for establishing thermal runaway processes and subsequent dynamic rupture propagation^51,52^. In this study, we are continuing the investigation by comparing the triggering conditions of these seismic ruptures and their focal mechanisms.

The volume changes associated with the dehydration of antigorite (the high-temperature serpentine) are expected to be around + 5% at a depth of 40 km (i.e. ≈ 1 GPa) and −1% at a depth of 140 km (i.e. ≈ 4 GPa)^53^. In addition, extensive experimental works on the antigorite stability field (e.g.^27,54^) have demonstrated that the Clapeyron slope of antigorite dehydration can significantly vary depending on various natural parameters^17^, leaving us uncertain as to their potential impact on the stress field. Consequently, we use thermodynamics in this paper to estimate the reaction volume change of antigorite dehydration by discussing relevant parameters and simplifications. The results of the stress inversion are presented hereafter (*Results*) and discussed in light of the new thermodynamic calculations (*Discussion*).

## Results

**Figure 2:**
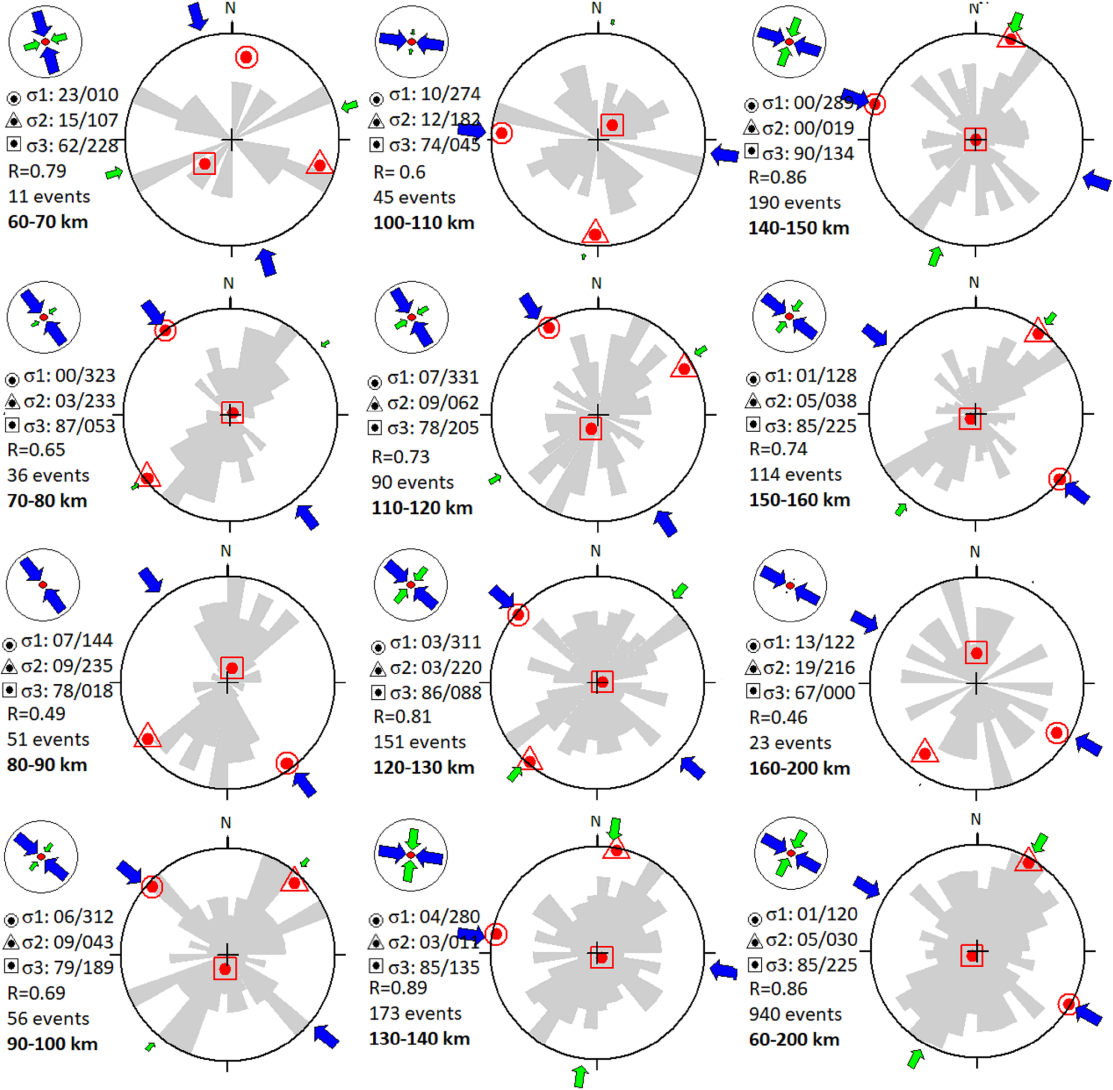
Focal mechanism stress inversion for different depth intervals (every 10 km). Stereograms showing the stress inversion results, including the strike direction of fault plane solutions (grey rose diagram), the stress ratio $R$ (defined in the *Methods*) and the number of focal mechanisms for each depth interval. The orientations of the three principal stress axes (plunge/azimuth) are represented with a red circle for ${\sigma }_{1}$, a triangle for ${\sigma }_{2}$ and a square for ${\sigma }_{3}$. The axes of the maximum and minimum horizontal stresses ${Sh}_{\mathrm{max}}$ and ${Sh}_{\mathrm{min}}$ are represented by blue and green arrows, respectively. Their type, length and colour symbolise the horizontal deviatoric stress amplitude relative to the isotropic stress (${\sigma }_{i}$), depending on the stress regime.

The stress field for the Vrancea intermediate-depth seismic body (60–200 km) is calculated using the inversion of 940 focal mechanisms for events that occurred between 1929 and 2020^22,23^. To observe the stress field evolution with depth (Fig. 2) and the associated distribution of focal mechanisms (Table S1; Fig. S1), we divided them into 10-km thick depth intervals.

The spatial distribution per depth interval does not show any significant horizontal variation of focal mechanisms along the NE-SW Vrancea zone (Fig. S1). The predominant faulting style for the analysed data (Vrancea subcrustal events) consists of reverse faulting (Fig. S1), with most nodal planes oriented in the NE-SW direction, i.e. parallel to the Carpathian Arc. However, a non-negligible amount of strike-slip and normal faulting, as well as oblique faulting, can be observed. An interesting feature of the subcrustal seismic body is the trend of the extension axis T and the compression axis P, almost vertical and horizontal, respectively (Fig. S2), that would be consistent with horizontal compression regardless of depth, except between 60 and 70 km due to insufficient data.

Stress inversion results (Fig. 2) clearly characterize the stress regime, in agreement with the tectonic framework of the Vrancea slab^55^. Seismic sources reveal a stress field consistent with an almost pure compressional stress regime (${\sigma }_{3}$ vertical) with directions of the maximum horizontal principal stress *Sh*_*max*_ ranging from NNW-SSE to WNW-ESE (Table S1, Fig. S2). The exception to the latter for the 60–70-km and 160–200-km intervals, are due to a number of focal mechanisms that is not sufficient to provide relevant results. Yet, the role of ${\sigma }_{2}$ should not be overlooked, as a horizontal compression does not necessarily reflect a collision setting but can also be associated to vertical extension due to slab pull for instance (e.g.^56–58^).

The amplitudes of the principal stress components are expressed in a relative manner because the absolute values cannot be determined using geological data only^59^, so the amplitudes of ${\sigma }_{2}$ is fixed by the stress ratio $R$ as a function of ${\sigma }_{1}$ and ${\sigma }_{3}$ (Fig. 2). At depths between 60 and 100 km, the stress field is purely compressional with a value of the stress ratio $R$ between 0.49 and 0.79 and a dominant NNW-SSE compression trend (Fig. 2). Around 100 km depth, the horizontal compression transitions into vertical extension between 100 and 140 km, with an increase in the stress ratio $R$ to 0.81 (Fig. 2; Table S1) and a rotation of the main compressive stress axes (Fig. 2; Fig. S2). In this depth interval, the Clapeyron slope of serpentine dehydration is negative, which could explain the vertical extension recorded by the intermediate-depth focal mechanisms (*Discussion*). Finally, at depths > 140 km, where the dehydration of the 10-Å phase (or other minerals such as phase A) is characterized by a positive Clapeyron slope^9^, focal mechanisms indicate a compressional stress field and $R$ decreases.

**Figure 3:**
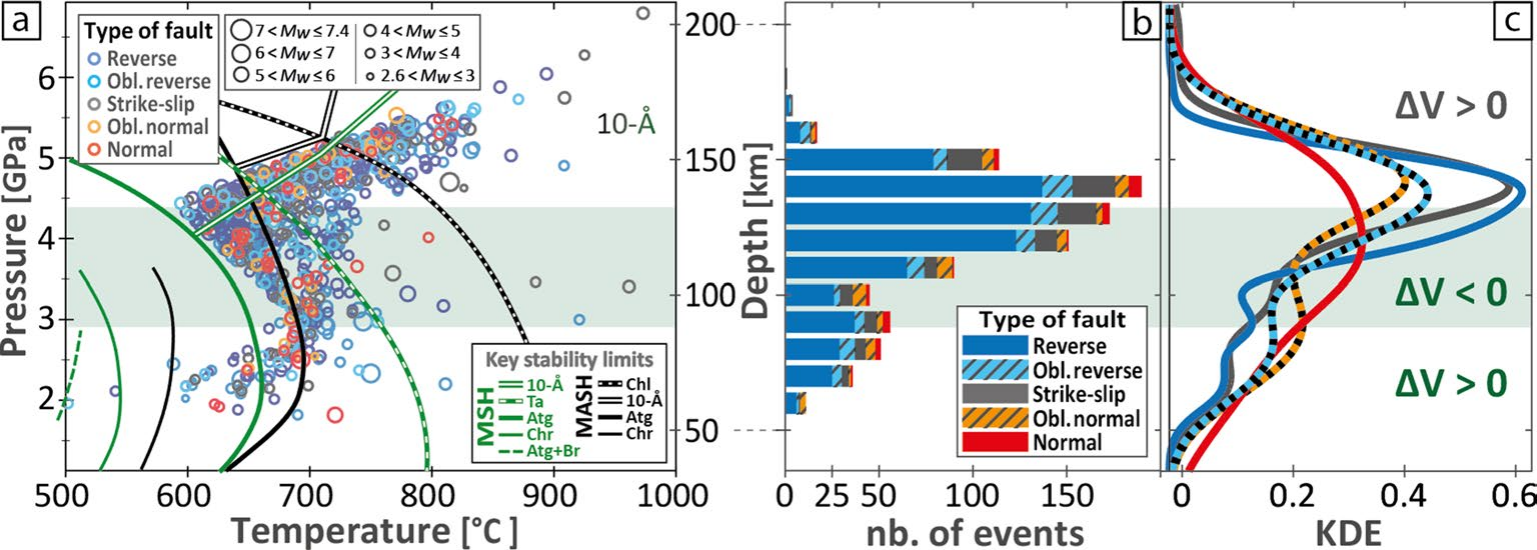
Mineral stability and distribution of seismicity in the Vrancea slab. (**a**) Pressure–temperature diagram showing conditions at hypocentres and the stability limits of key minerals^9^; (**b**) depth distribution of the seismicity; (**c**) kernel density estimation (KDE) of the earthquake’s dataset for each focal mechanism. Oblique reverse faults and oblique normal faults respectively correspond to reverse and normal faults with substantial stricke-slip components. The green shade accounts for serpentine dehydration with a negative Clapeyron slope. Extended versions of **a** and **b-c** are presented in Figs. S3 and S4, respectively, showing the distribution for each class of focal mechanism. Abbreviations of minerals: Atg = antigorite; Br = brucite; Chr = chrysotile; Chl = chlorite; Ta = talc; 10-Å = 10-Å phase. Simplified chemical systems: MSH = MgO–SiO_2_–H_2_O; MASH = MgO–Al_2_O_3_–SiO_2_–H_2_O.

Overall, the Vrancea region is characterized by a regional NW–SE compression and vertical extension within the slab^60^. Vertical extension dominates if we proceed to a stress inversion for the entire 60–200-km depth interval (Fig. 2), although mostly expressed in the form of reverse faulting (Fig. 3). We confirm that the compressional setting is recorded by the distribution of focal mechanisms at depths between 60 and 100 km and > 140 km, i.e. depth ranges characterized by dehydration reactions with positive Clapeyron slopes (Fig. 3). However, we highlight that vertical extension recorded by intermediate-depth focal mechanisms between 100 and 140 km correlates with the negative Clapeyron slope of serpentine dehydration in this depth interval (Fig. 3), suggesting that the signal observed between 100 and 140 km should be considered as an anomaly.

## Discussion

### A simple signal thanks to a simple and static geometry

While the subduction of the Pacific Plate beneath Northern Japan can be considered, to some extent, as a natural laboratory to study active subduction processes^26,36,61^, we can see the relatively simple and static geometry of the Vrancea slab as a natural laboratory that enables the deconvolution of contributions from tectonic forces and intrinsic slab properties in the observed seismicity^9^. The limited delamination of the Carpathian lithosphere^20^ that induced slab verticalization^4,62^ has resulted in a relatively simple slab geometry, which helps the visualization of the potential impact of dehydration reactions on the stress field amplitude and orientation.

**Figure 4:**
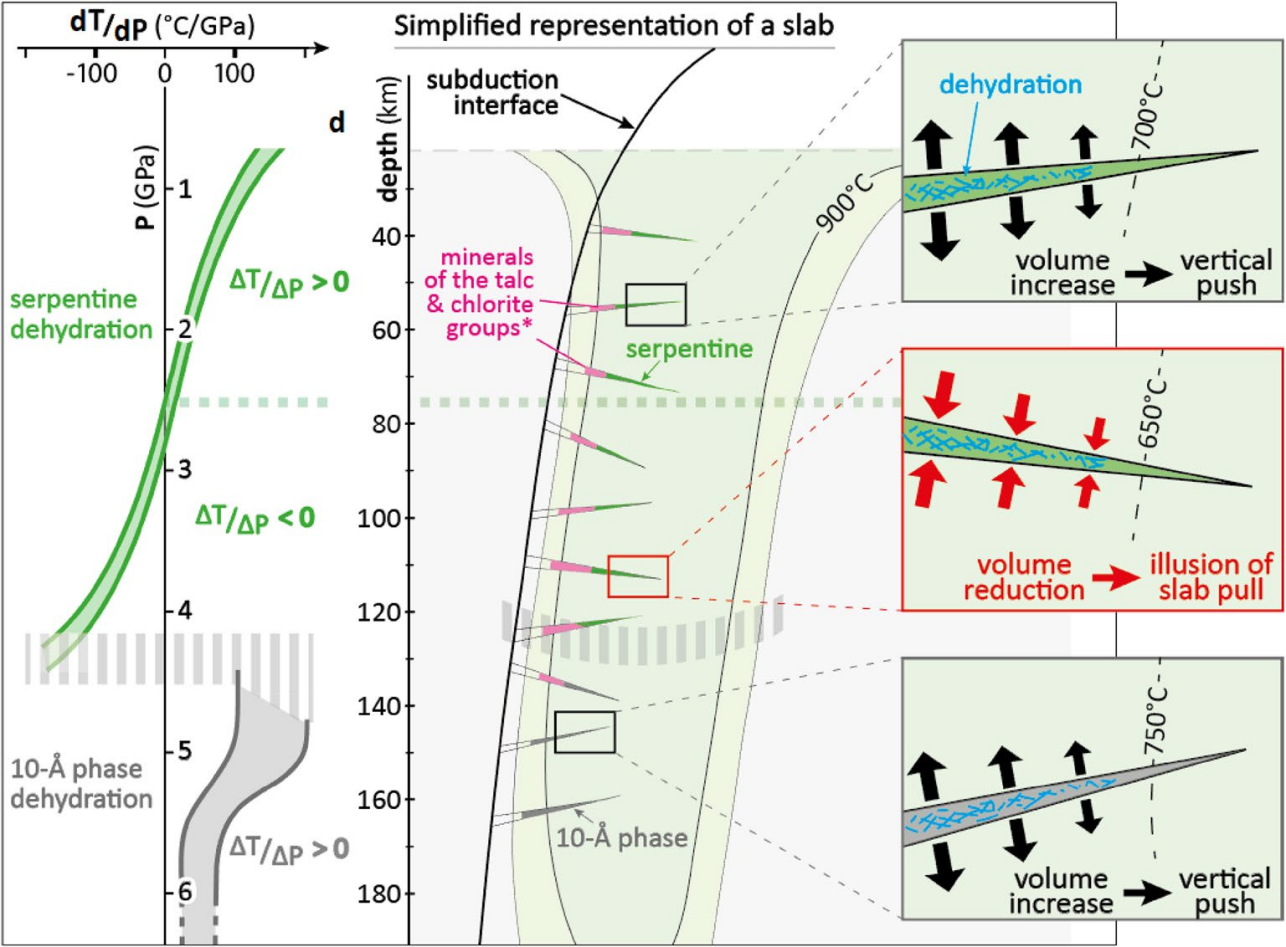
Stress field evolution with volume change of dehydration reactions within the slab. (**a**) Depth evolution of the inverse Clapeyron slope for the main mineral dehydrations related to the Vrancea seismicity^9^; (**b**) expected distribution of the serpentinized faults^17^ within the slab. Positive or negative Clapeyron slopes imply positive or negative total volume changes (solid + fluid), respectively, which impacts the stress field within the surrounding peridotites, as reflected by the focal mechanisms of earthquakes occurring within the latter.

At first order, the maximum and minimum horizontal stresses *Sh*_*max*_ and *Sh*_*min*_ respectively align with ${\sigma }_{1}$ and ${\sigma }_{2}$, and ${\sigma }_{3}$ is vertical (Fig. 2). Nonetheless, the force balance responsible for the seismic distribution within the Vrancea slab clearly switches to vertical extension at ≈ 100 km and switches back to the signal of horizontal compression at ≈ 140 km. Contrary to active subductions, only one parameter (temperature) is expected to evolve in the locked Vrancea slab^9^. The subducted panel of lithospheric mantle can be simply considered as volumes of fresh peridotite regularly separated by serpentinized faults^14,17^. While at first order the stress field is explained by Newton's laws and the density distribution within the studied volume, key heterogeneities can significantly modify the stress field between them^16^. As illustrated on Fig. 4, we propose that the volume reduction occurring upon serpentine dehydration in this depth range is directly responsible for the observed change in the stress field between 100 and 140 km.

### The careful use of the Clapeyron equation and numerical predictions in a natural context

For a given transformation, the evolution of the reaction pressure *P* as a function of the temperature *T* is directly related to the ratio between the enthalpy of transformation ${\Delta H}_{\mathrm{r}}$ and the associated volume change $\Delta V$ as follows:

$$\frac{dP}{{dT}} = \frac{{\Delta H_{{\text{r}}} }}{T \cdot \Delta V}$$

In a P–T diagram, the Clapeyron slope (d*P*/d*T*) indicates the sign of the expected volume change (ΔV), but only in specific conditions. Positive and negative d*P*/d*T* are associated with positive and negative ΔV (solid + fluid), respectively (Fig. 3), assuming no fluid escape. Dehydration reactions have a positive ${\Delta H}_{\mathrm{r}}$, thus positive d*P*/d*T* are associated with positive ΔV (solid + fluid), potentially inducing fluid overpressure, whereas a negative d*P*/d*T* implies a negative ΔV (solid + fluid), prone to local stress release or pressure drops around the transforming volume^14,46^. In Fig. 4 we use the inverse Clapeyron slope (d*T*/d*P*) in order to get a linear function with no asymptotic behavior. The anomaly of the stress field highlighted in the present paper correlates with the Clapeyron slope of the main dehydration reaction controlling the seismicity^9^. Our main findings are summarized in a synthetic cross-section (Fig. 4) showing the depth correlation between the d*T*/d*P* of the main reactions and the stress field orientation. The Quaternary verticalization of the Vrancea slab implies that the bending faults formed within this segment of Alpine Tethyan lithosphere should be sub-horizontally distributed in the present-day configuration. This simple geometry makes the results relatively straightforward, as well as the link between triggering and focal mechanisms.

Between 100 and 140 km depth, the Vrancea seismicity is demonstrably related to the high-pressure dehydration of antigorite^9^, characterized by a negative d*T*/d*P* at these depths (Fig. 4; Fig. S5), alike other reactions involving minerals of the serpentine, talc or chlorite families^27^. Because of negative d*T*/d*P* between 3 and 4.5 GPa, local overpressure cannot be the cause for embrittlement^46,48^. We confirm that the stress field is incompatible with a mechanism based on overpressure in a closed system^14^. In contrast, hereafter we propose that self-sustained fluid percolation events, although not necessarily continuous in time and space, could cause enhanced volume reduction that would contribute to the observed stress field anomaly.

Importantly, it should be highlighted that, whereas thermodynamic modelling well reproduces experimental data at low pressures (< 2.5 GPa, i.e. < 75 km), a serious discrepancy remains at high pressures (Fig. S5^54^). Pseudosections based on thermodynamic equilibrium and mass balance calculations (e.g.^63^) allow to model simplified systems (Figs. S6 and S7) and to predict the evolution of these systems upon (slow) heating (Fig. S8). The latter are calibrated thanks to experiments (e.g.^54^). However, considering natural variabilities in mineral chemistry, fluid saturations and other parameters affecting parageneses stability^17^, the Clapeyron slope of serpentine dehydration can significantly vary at high pressures, especially depending on its Al^3+^ content^17,64^. For pressures between 4 and 4.5 GPa, serpentine destabilization reactions in the up-to-date knowledge (see^17^ for a review) exhibit d*T*/d*P* varying from −30 to −230 °C/GP (Fig. 4), which could correspond to ΔV (solid + fluid) varying between −1 and −5%^53^.

Pseudosections are associated with uncertainties that increase with increasing pressure, and the results of the calculations depend on the a priori knowledge about the mineral phases that can exist in the studied conditions^17^. To date, no thermodynamic data exists regarding the dehydration of the 10-Å phase or any other high-pressure hydrated talc, thus making difficult the mass balance calculation for the dehydration around 4 GPa. Nonetheless, data exist on phase A, which dehydrates in almost the same P–T conditions as the 10-Å phase and exhibits a similar positive Clapeyron slope (and thus a similar reaction volume change). Allowing phase A to exist in the numerical model (Fig. S7) significantly increases the apparent d*P*/d*T* of antigorite dehydration (Fig. S6), partly due to the partial dehydration of antigorite that forms phase A at high pressure^54^. The addition of the 10-Å phase in future numerical simulations should allow a full match with the Vrancea seismicity. Other parameters such as H_2_O saturation, natural chemical variability, fluid percolation and brucite availability are also important parameters influencing the Clapeyron slope and associated volume change.

### Shallow pervasive serpentinization vs localized self-propagating corrosion of fault roots

At shallow depths, the volume increase induced by serpentinization can reach up to > 40%^65^, generating stresses sufficient to reach the tension rupture criterion in olivine, leading to full cracking of olivine grains from which the mesh texture of serpentine veins originates (e.g.^32^). In addition, the exothermic nature of the serpentinization reaction enables to maintain relatively high temperatures (200–300 °C) in the vicinity of cracking olivine grains, which contribute to efficient pervasive serpentinization, as widely reported for the first kilometres below the oceanic floor (e.g.^32^). In contrast, at high pressures (> 1 GPa), efficient reaction front migrations such as pervasive serpentinization are inhibited (transdisciplinary discussion^17^). Initial cracking or strain localization processes are required to allow local fluid percolation, as proposed as a feed for deep serpentinization of fault roots^17,26,30,33,66^. Consequently, the ability of (de)serpentinization reactions (among others) to generate their own vein network and self-propagation via reactive porosity^41^ is a key discovery that helped us understand how deep serpentinization could occur before subduction^26,30–33^. Additionally, the internal nanoporosity of serpentine minerals give them the ability to become volatiles pathways enabling further hydration/dehydration reactions with evolving P–T conditions^17^. In addition, metasomatic reactions can control the fate of dehydration fluids, and may strongly influence fluid pathways^67^.

Fluid mobility under high pressures and temperatures appears as a key parameter in (de)hydration reactions and metasomatic processes in general^41,42,67,68^. Importantly, water/protons mobility is not limited to fluid percolation processes. Volatiles can migrate through the seismic volume via reaction fronts migrations, i.e., either (de)hydration reactions or other metasomatic reactions^41,67^. Volatiles can be transferred from a phase to another with increasing pressure and/or temperature, e.g. antigorite to high-pressure hydrated talc phases^17^. In addition, at temperatures of 500 °C or higher, and pressures above 1 GPa, dehydration fluids, which can transiently exist as free fluid upon dehydration^43^ would consist of supercritical fluids^17^. Such corrosive fluids would not be stable for long and would react relatively fast with the surrounding rocks, forming new hydrous (and/or carbonate) rocks in the vicinity of the dehydration location.

### Closed system versus open system allowing fluid escape

The ability of fluids to migrate though connected fluid pathways would play an important role on the amplitude of the stress perturbation. Both mechanical instabilities triggering earthquakes and rupture-size stress distortions can be generated by dehydration, but these processes are not straightforward and, importantly, proceed at very different time and space scales. One could consider that d*T*/d*P* directly informs about the magnitude of ΔV and about the likelihood that the dehydration would induce mechanical instabilities. However, considering the dehydrating tip of a serpentinized fault as a closed system, numerical simulations show that only limited ΔV are expected, from about + 2% at 1 GPa to about −2% at 5 GPa (Fig. S9), which is unlikely to trigger a slab-scale change of the stress field. But in nature, serpentinized faults are open systems, with preferred fluid percolation paths within the clusters of serpentine patches distributed along the faults, via either reactive porosity or intrinsic nanoporosity of serpentine crystals^39–42,67,68^. Consequently, if fluid is allowed to escape (Fig. 4), ΔV will lie between the solid-only volume change and the closed-system volume change. As highlighted on Fig. S9, regardless of pressure, with either positive or negative Clapeyron slopes, the solid density change is positive and the associated ΔV is negative, up to nearly −20%. In addition, between 100 and 140 km depth, an initial volume change of −2% at the onset of dehydration may generate transient porosity favoring further fracturing and associated fluid percolation. Such runaway process would favor further negative volume change of reaction and a long-term impact on the stress field. The expected ΔV of 10–20% are definitely sufficient to induce mechanical instabilities around the dehydrating tip of serpentinized faults^9^ and we argue that this could also account for the stress field anomaly observed between 100 and 140 km depth.

In other words, we propose that the negative volume change enhances further dehydration and fluid escape by favouring the transient existence of connected fluid pathways. In contrast, the probability to form such fluids/volatiles pathways should be much lower within regions enduring positive volume changes, where dehydration fluids and/or hydrous/transforming materials (able to conduct volatiles) are expected to be significantly less connected. It is important to recall that the yield strength in tension is three times lower than its equivalent in compression, which is expected to increase seismicity and associated transient crack connectivity in case of negative volume changes^14^. Considering these different parameters altogether, the probability for high-efficiency fluid percolation processes should drastically increase as a result of the switch from positive to negative volume change at depths around 100 km.

### Anisotropic volume change and apparent slab pull

Certain subduction zones, such as the Ryūkyū-Kyūshū region^57^, are characterized by horizontal switches from slab pull to coupling along the subduction interface, which is attributed to either rheological or geometrical complexities of the latter^69–71^. Stress field inversions for active subducting slabs can exhibit changes between slab coupling and slab pull^72^, which could reveal variable rheological responses of the subduction interface with depth. The absolute slab pull component of the stress field is observed to increase with increasing seismicity^72^. In contrast, at depths > 140 km the Vrancea seismicity continues to increase while the apparent slab-pull signal switches back to horizontal compression (Figs. 2 and 3). A contribution from the positive buoyancy characterizing the deep half of the Vrancea slab could participate to actual slab pull^9^, but the stress field recorded by the seismicity at depths > 140 km is in contradiction with slab pull.

On one hand, as illustrated on Fig. 4, considering the simple geometry of the Vrancea slab, potential slab pull would result in a vertical extension consistent with the signal recorded between 100 and 140 km by focal mechanisms. On the other hand, volume change can have a significant impact on the stress field and even trigger seismic ruptures (e.g.^45,73^). While considering a closed system with no fluid escape leads to ΔV (solid + fluid) not exceeding -5%, natural conditions expected for the sub-horizontal serpentinized faults within the Vrancea slab can reach 10 to 20%. Considering the fast kinetics of such dehydration reactions^27–29^ and the positive feedback between shear heating and dehydration ^74^, such volume changes are likely to induce large stress amplifications, consistently with field observations (e.g.^15,50^). Numerical modelling shows that local rheological contrasts can generate stress accumulations up to several gigapascals^16^, as confirmed by experimental studies under synchrotron radiation^14,75^ and consistent with field observations^15,50^. These stress accumulations are expected to be augmented upon mineral destabilizations^14,17^. Thanks to the above-mentioned self-sustained dehydration process with negative ΔV (solid + fluid), the serpentine dehydration is therefore expected to trigger significant vertical extension between 100 and 140 km, explaining the observed apparent “slab pull”.

### Stress field rotation and slab detachment

Slab detachments can be imaged in certain regions, such as Hindu Kush (i.e.^58^), which requires substantial data and adequate imaging techniques. We show that the “slab pull” signal deduced from the stress inversion of focal mechanisms is not sufficient to conclude for a slab detachment. Most subduction zones appear more complex than Vrancea, with oblique geometry and coeval evolution of pressure, temperature, stress, strain and fluid percolation, making the interplay between these parameters difficult to unravel.

As actively subducting slabs endure subduction, mineral destabilizations occur as pressure and temperature gradually increase. At any depth, there is a dominant stable phase to which the released volatiles (e.g., H_2_O, CO_2_) can be transferred^17^. Reaction after reaction, some key mineral reaches significant rock fractions in certain P–T windows, such as glaucophane within the metamorphized oceanic crust or antigorite within the uppermost mantle. Several mineral destabilizations have significantly negative Clapeyron slopes at given depths, such as, for instance, serpentine minerals between 100 and 140 km depth.

The rotation of the stress field that accompanies the switch from horizontal compression to vertical extension likely favours both fault reactivation and fluid percolation. Such switches in the stress field due to switches between positive and negative Clapeyron slopes could trigger strain localization events leading to repeated weakening of the slab, further favouring slab detachment. Consequently, localized slap-pull signals, while not indicating that any external force would be pulling the slab, could be an indicator of internal slab transformations involved in the slab detachment process of actively subducting lithospheric slabs.

## Conclusions

The parallel investigation about triggering mechanisms and focal mechanisms in the Vrancea slab highlights the central role of serpentine minerals in controlling intermediate-depth seismicity. The analysis of the stress field reveals a switch from horizontal compression to vertical extension between 100 and 140 km depth, where the Clapeyron slope of serpentine dehydration is negative. This constitutes a stress field anomaly that one could interpret as “slab pull”.

The simple geometry of the verticalized Vrancea slab, with sub-horizontal serpentinized faults, helps to understand stress fields in subducting lithospheres. The negative volume change of the dehydration reaction and the ability of dehydration fluids to escape the system via reactive porosity can generate a volume reduction of 10 to 20%, i.e. ten times higher than assuming a closed system. Consequently, we argue that the correlation between the stress field anomaly and the negative volume change is no chance. Serpentine dehydration in the sub-horizontal serpentine patches would induce a significant perturbation of the stress field, i.e. local vertical extension recorded by the intermediate-depth seismicity.

We conclude that the seismological signal that could be interpreted as “slab pull” between 100 and 140 km within the Vrancea slab can be explained by the volume reduction within a sub-horizontal set of serpentinized faults. This would mean that efficient percolation paths exist within the Vrancea slab, allowing dehydration fluids to laterally escape, certainly towards the former subduction channel. In other words, depending on how protons fit in the structure of minerals at depth, dehydration reactions have very different consequences on the stress field, which for instance would explain the switch from horizontal compression to vertical extension.

Combined with a previous study^9^, the present paper evidences that dehydration within the Vrancea slab induces both (1) local mechanical instabilities triggering earthquakes and (2) stress field distortions at the scale of the peridotite volumes located in between the serpentinized faults, as recorded by focal mechanisms. These two processes do not occur at the same time and space scales.

This apparent “slab pull” is accompanied with a rotation of the main compressive stress. Such dehydration-induced perturbation of the stress field could favour strain localization. Repeating rotations, e.g., back to a compressional setting at 5 GPa, would generate substantial weakening via fault reactivation and stress corrosion following percolation paths, promoting slab detachments in actively subducting slabs.

## Methods

### Focal mechanisms and stress field inversion

To better grasp the current seismogenic stress fields, we compiled an up-to-date catalogue including 940 available focal mechanisms for the studied area. The events have moment magnitudes *M*_W_ > 2.7 and occurred between 1929 and 2020. We estimate 437 focal mechanism solutions for events since 2005^22^; older data are from the REFMC catalogue^23^.

Since 2004, a significant number of new seismometers have been deployed by the Romanian Seismic Network (RSN, https://doi.org/10.7914/SN/RO), which increased the number of detected seismic events affecting the Romanian territory ^76^. As a consequence, the magnitude threshold for which focal mechanisms can be determined for intermediate-depth earthquakes dropped from *M*_W_ 4 to *M*_W_ 2.7^11^.

The fault plane solutions are obtained using the FOCMEC code^77^ using the velocity model of^7^ and integrated in the SEISAN software^78^. These solutions were estimated from P-wave polarities for events with at least 10 records, filtered based on the weight assigned to the P onset (discrete classed from 0, best, to 3, worse) keeping only polarities for picks with weights 0 and 1 and rejecting those with weight 2 and 3, therefore solutions' confidence level is reliable^79^. The location of the seismic stations and seismic events can be seen in Fig. 1. To ensure a good quality of the dataset and a high confidence level in the computation of the focal mechanism solutions, we visualized and manually picked the employed waveforms. Primary information about the seismic events (as location and *M*_W_) is taken from^80^ and the ROMPLUS catalogue^81^.

The retrieved focal mechanisms are presented in Fig. 2 along with the pre-2005 dataset. The depth distribution and classification of intervals are shown in Fig. S1 (*Suppl. Information*), with different colours corresponding to different focal mechanisms according to the standard classification used in the World Stress Map^82^ and based on the dip angles of the T, B and P axes^83^. We apply an orientation criterion distinguishing reverse faults, normal faults, strike-slip faults and oblique mechanisms (Table S1). Reverse faults with SW-NE and NW–SE strikes, i.e., respectively parallel and normal to the Vrancea seismic body, are presented separately in Figs. S3 and S4.

The stress regime was determined from the inversion of the focal mechanisms using the *Win TENSOR* software^59^. The fault-slip data are inverted to obtain the reduced stress tensor parameters, i.e. the principal stresses ${\sigma }_{1}$, ${\sigma }_{2}$ and ${\sigma }_{3}$ and the stress ratio $R=({\sigma }_{2}-{\sigma }_{3})/({\sigma }_{1}-{\sigma }_{3})$, which defines the magnitude of ${\sigma }_{2}$ relative to ${\sigma }_{1}$ and ${\sigma }_{3}$ (0 < $R$  < 1). A high $R$ means that ${\sigma }_{2}$ tends to equal ${\sigma }_{1}$ (extension along the ${\sigma }_{3}$ axis), while a low $R$ indicates that ${\sigma }_{2}$ and ${\sigma }_{3}$ exhibit close values, i.e. pure collision setting^84^.

The horizontal principal stress directions *Sh*_*max*_ (maximum horizontal stress) and *Sh*_*min*_ (minimum horizontal stress) are calculated using the method of^85^. The stress inversion results for each depth interval are presented in Fig. 2 and the distribution of the principal stress axes of the focal mechanisms (P-compression and T-extension axes) are shown in Fig. S2. The depth distributions of *Sh*_*max*_ and $R$ are shown in Fig. S5. A high $R$ value for a compressional regime means that *Sh*_*max*_ (// ${\sigma }_{1}$) and *Sh*_*min*_ (//${\sigma }_{2}$) are not very well differentiated.

### Comparison between focal mechanisms and triggering mechanisms

To evaluate the link between earthquake triggering mechanisms and focal mechanisms in the Vrancea slab, the pressure–temperature (P–T) diagrams were built for each type of focal mechanism (Fig. S3). For each hypocenter, the pressure was computed from the 3D P-wave velocity structure^86^ using the procedure proposed by Ferrand & Manea^9^, while the temperature was extracted from the most recent 3D thermal model^21,87,88^.

The uncertainties on these P–T conditions are described by^9^. Based on a review of experimentally-deduced stability limits for the mineral phases expected in the slab^9,17^, the P–T conditions for each hypocenter are presented accompanied with key dehydration reactions in Fig. 3, along with the distribution of focal mechanisms with depth and the corresponding kernel density estimation (KDE^89,90^).

### Clapeyron slope, volume change and apparent slab pull

Our estimates of volume change during dehydration of serpentinite are based on thermodynamic equilibrium calculations for the breakdown of antigorite. The following reaction may be used to describe the dehydration of antigorite in the pure Mg, Si, O, H system:

1 $${\text{Mg}}_{48} {\text{Si}}_{34} {\text{O}}_{85} \left( {{\text{OH}}} \right)_{62} = 10 {\text{Mg}}_{2} {\text{Si}}_{2} {\text{O}}_{6} + 14 {\text{Mg}}_{2} {\text{SiO}}_{4} + 31 {\text{H}}_{2} {\text{O}}$$

This reaction can be plotted in P–T space (Fig. S6), using the Gibbs energies of the phases antigorite, forsterite, enstatite and water calculated with *Thermolab*^63^ and the dataset of^91^. The reaction with the pure phases shows a reasonable fit to the experimental data from^54^ at low pressure, but not at high pressure. Additionally, the data points at higher temperatures lie in the domain where all antigorite is already reacted out, however the experimental datapoints still contain antigorite.

In natural rocks, pure phases are uncommon, and antigorite can incorporate significant amounts of Fe, and Al. The experimental data points also involved Al and Fe bearing antigorite. We therefore selected a composition of antigorite solid solution which includes several weight percent of Al and Fe. Here the solid solution is modeled according to^92^. Other phases involved in the computation were olivine, orthopyroxene, from^93^, talc from^94^, brucite taken as an ideal binary Fe–Mg solution, and phase A was treated as pure phase. The equation of state for water^95^ was used in accordance with the^91^ dataset. Involving solid solutions, univariant reactions become multi-variant fields (Fig. S7). Within such fields the dehydrating phases disappear over a range of P and T (Fig. S8). Furthermore, the curvature of the onset of antigorite breakdown (Fig. S8) fits better to the experimental data (Fig. S8). The experimental points between 2 and 4 GPa lie in the domain where already olivine is formed but some antigorite is still left, which is also the case for the experimental run product of those points. The density of the solid changes as a function of P–T caused by the different dehydration reactions. A strong density increase of the rock is associated with this reaction. This can be expected when a hydrous assemblage transforms into an anhydrous rock.

The system density is given by:

2 $$\rho_{sys} = \rho_{s} \cdot \left( {1 - \varphi } \right) + \rho_{f} \cdot \varphi$$

where ${\rho }_{s}$ is solid density (plotted in Fig. S7b), and ${\rho }_{f}$ the fluid density, and $\varphi$ is porosity.

Densities of fluid and solid are calculated from thermodynamic data but porosity is not constrained from thermodynamics. The following mass balance equation has been used to calculate the porosity:

3 $$\frac{{\partial C_{s} \rho_{s} }}{\partial t} + \frac{{\partial \left( {\,\rho_{s} \cdot \left( {1 - \varphi } \right) \cdot C_{s} } \right)}}{\partial x} = 0$$

where ${C}_{s}$ is the concentration of an immobile species in the solid. Equation (3) is the mass conservation of one of the components in the solid that is conserved, so it is remaining in the solid after dehydration. The only assumption here is that solid is not deforming and not moving. Integrating this with time and solving for porosity as is shown by^68^:

4 $$\varphi = 1 - \frac{{\rho_{s0} \cdot \left( {1 - \varphi_{0} } \right) \cdot C_{{s_{0} }} }}{{\rho_{s} \cdot C_{s} }}$$

where the subscript 0, indicates that these are initial values of the density and concentration. With (2) and *(4)* the system density is obtained after choosing a particular initial porosity, solid density and concentration. Because we are interested in the solid density and volume changes during the dehydration, this point is taken at a fixed pressure and the starting temperature at the onset of dehydration. Three different cases are shown in Fig. S9: a transect through temperature at 1 GPa, 4 GPa and 5 GPa. In all three cases the solid density change is positive, and the associated volume change is negative reaching up to nearly −20%. The total system volume change is negative at 4 and 5 GPa and is positive at 1 GPa. This reflects the change in slope of the reaction. A maximum of about −2% (negative system volume change) is achieved in the case that all fluid is kept in the system. If fluid is allowed to escape the volume change will lie between the two extremes: (1) the pure solid volume change and (2) the system volume change assuming not fluid flow. At the onset of dehydration, the initial negative volume change of 2% may generate porosity, and it may lead to fracturing creating possibility of fluids to escape. This then leads to further negative volume change of reaction.

The current numerical simulation shows that including the possibility of high-pressure hydrated phases in the system is a key of the observed seismicity, thus reproducing better the natural conditions that would explain the switch in the stress field. In the future, we expect a better fit with including the stability limit of high-pressure hydrated talc phases such as the 10-Å phase^17^.

## Supplementary Information

Supplementary Information.

## Data Availability

The raw seismological dataset is accessible at 10.17632/tdfb4fgghy.2 and the catalogue of focal mechanisms is accessible at 10.17632/mykkx4gygy.3.
