# Supplementary material for: A switch from horizontal compression to vertical extension in the Vrancea slab explained by the volume reduction of serpentine dehydration
Source: Sci Rep. 2022 Dec 24;12:22320. doi: 10.1038/s41598-022-26260-5 (PMC9789975; doi:10.1038/s41598-022-26260-5)
Supplement: Supplementary file 1 — Supplementary Information. [file 41598_2022_26260_MOESM1_ESM.pdf]

A switch from horizontal compression to vertical extension in the Vrancea slab explained by the volume reduction of serpentine dehydration

Craiu, A.[1], Ferrand, T. P.[2,*], Manea, E. F.[1,3], Vrijmoed, J. C.[2] & Mărmureanu, A.[1]

[1] National Institute for Earth Physics, Calugareni, 12, Măgurele, Ilfov, Romania;
[2] Institut für Geologische Wissenschaften, Freie Universität Berlin, Malteserstraße 74-100, Berlin 12249, Germany;
[3] GNS Science, PO Box 30-368, Lower Hutt, New Zealand.
*corresponding author: thomas.ferrand@fu-berlin.de

Supplementary Information

Table S1 | Depth distribution of focal mechanisms and Clapeyron slopes of dehydration. R = Reverse faults (a = aligned; n = normal); S = strike-slip faults; N = normal faults. RS and NS = mixed mechanisms (oblique fault slip). The horizontal principal stress Sh and stress ratio R are described in section ***Methods***. The orientation $Sh_{\min}$ is normal to $Sh_{\max}$. The value of *R* for the entire dataset (60 to 200 km) is not the average of the row but the calculated stress ratio considering all events, which is largely influenced by the high seismic activity in the 120-150-km depth range.

Depth interval	Earthquakes sorted focal mechanisms								Azimuth of max. horizontal stress and stress ratio		main seismicity trigger	Clapeyron slope of the main reaction
	**all**	**R**	**Ra**	**Rn**	**RS**	**S**	**NS**	**N**	***Sh*$_{\max}$ [^{0}]**	***R***		
60-70	11	6	3	3	1	1	0	3	164	0,79	antigorite	> 0
70-80	36	25	17	8	5	4	1	1	143	0,65		
80-90	51	29	19	10	8	6	3	5	143	0,49		
90-100	56	37	23	14	5	7	4	3	130	0,69		< 0
100-110	45	26	14	12	3	7	2	7	97	0,60		
110-120	90	65	42	23	9	7	1	8	148	0,73		
120-130	151	123	69	54	10	12	1	5	132	0,81		
130-140	173	131	72	59	14	21	4	3	98	0,89		
140-150	190	137	69	68	16	23	7	7	109	0,86	10-Å phase +/- phase A	> 0
150-160	114	79	55	24	7	19	3	6	128	0,74		
160-200	23	10	3	7	6	4	1	2	119	0,46		
**60-200**	**940**	**668**	**386**	**282**	**84**	**111**	**27**	**50**	**120**	**0,86**		

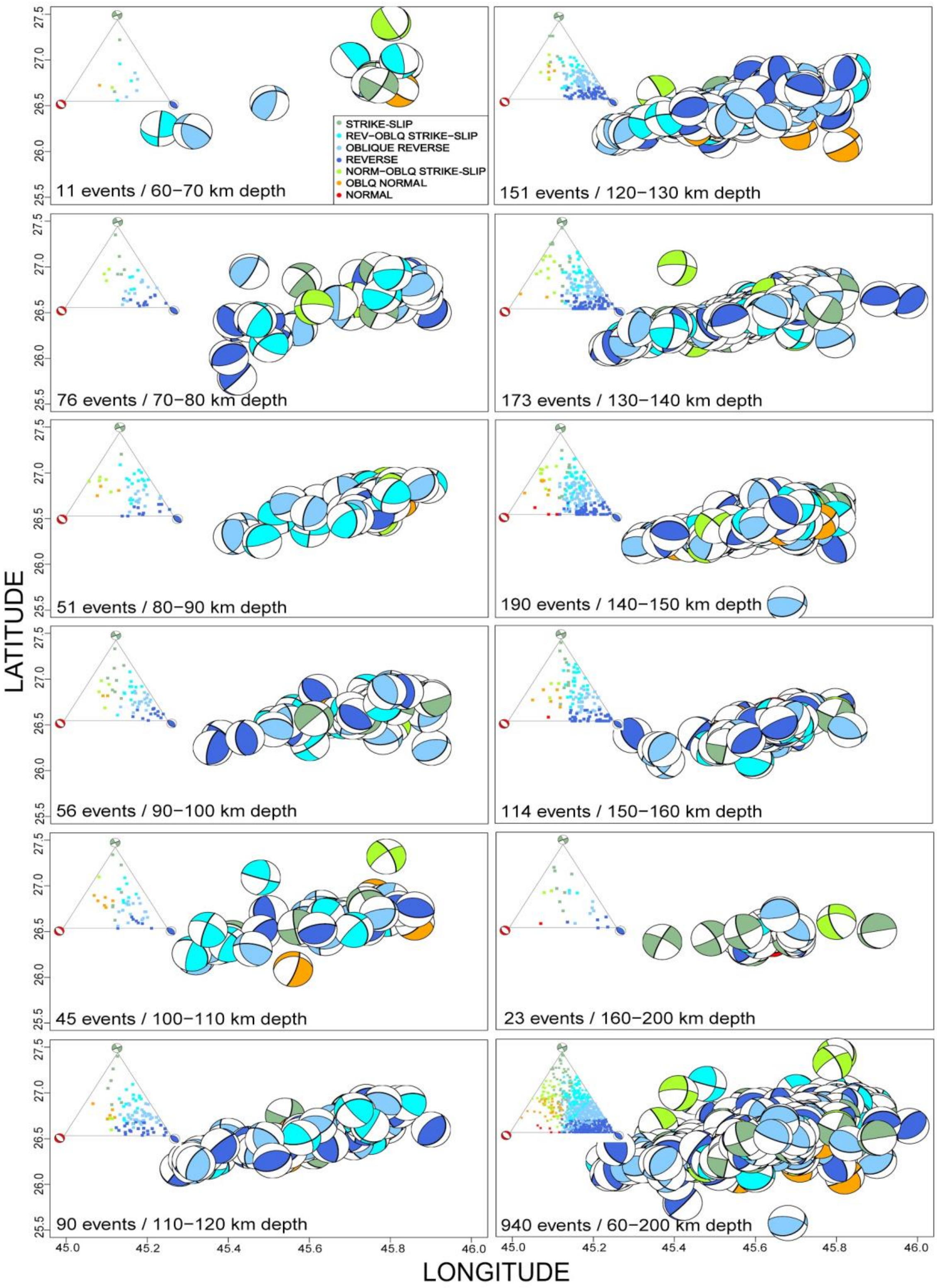

Figure S1 | Focal mechanisms spatial distribution of the Vrancea seismic body. Ternary diagram of focal mechanisms (different faulting styles), with selected number of focal mechanisms for each depth interval. The type of focal mechanism is displayed in legend, with different colours corresponding to different types of faulting according to the standard classification (Frohlich, 1992). The dataset of 940 events for the 1929-2020 period is presented in **Table S1**.

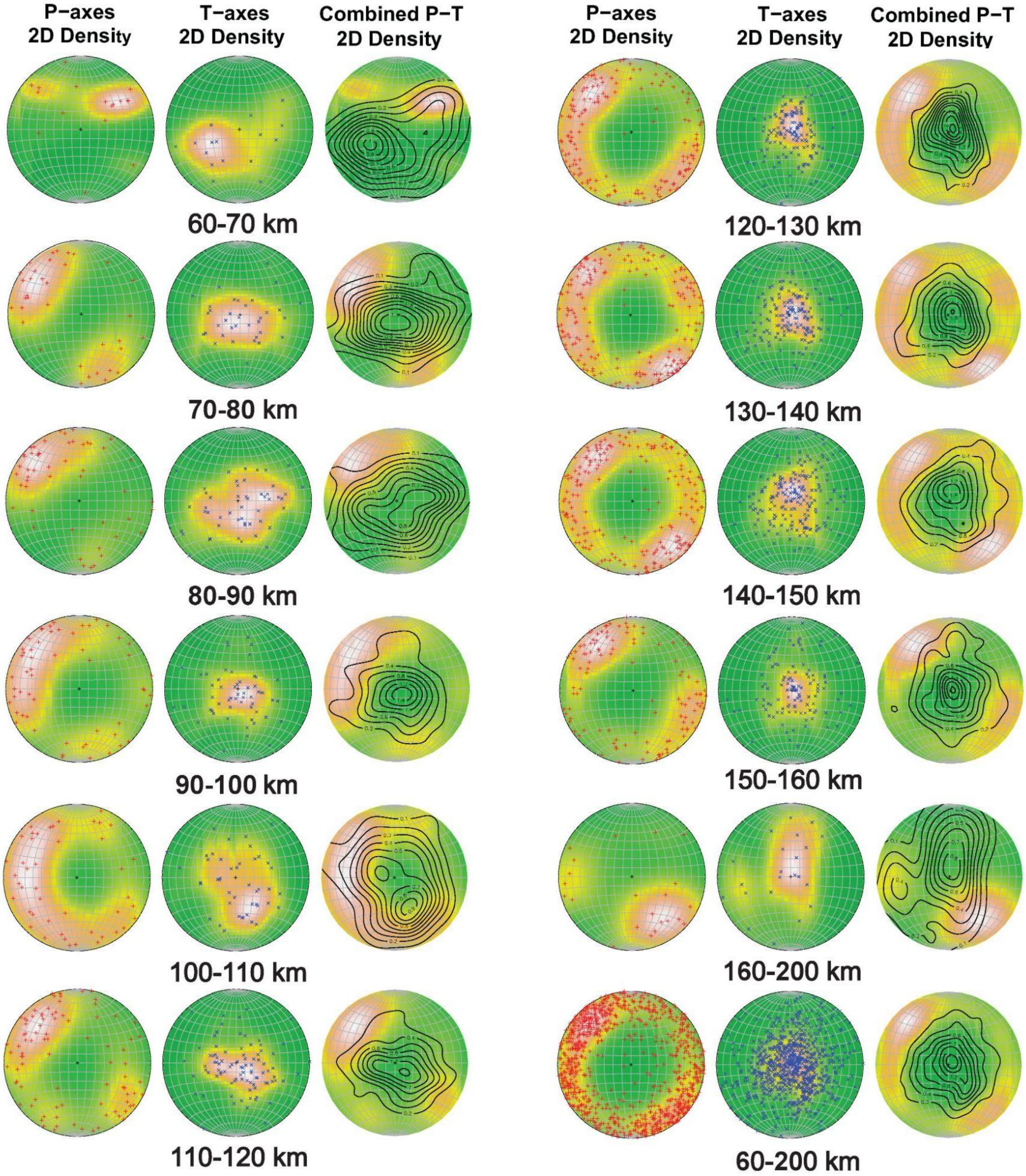

Figure S2 | Results of the stress inversion. Stereographic projections of the compression (P), extension (T) axes and combined of focal mechanisms for the period 1929-2020. Black dots show individual focal mechanism solutions. The coloured background shows the smoothed density distribution of all solutions.

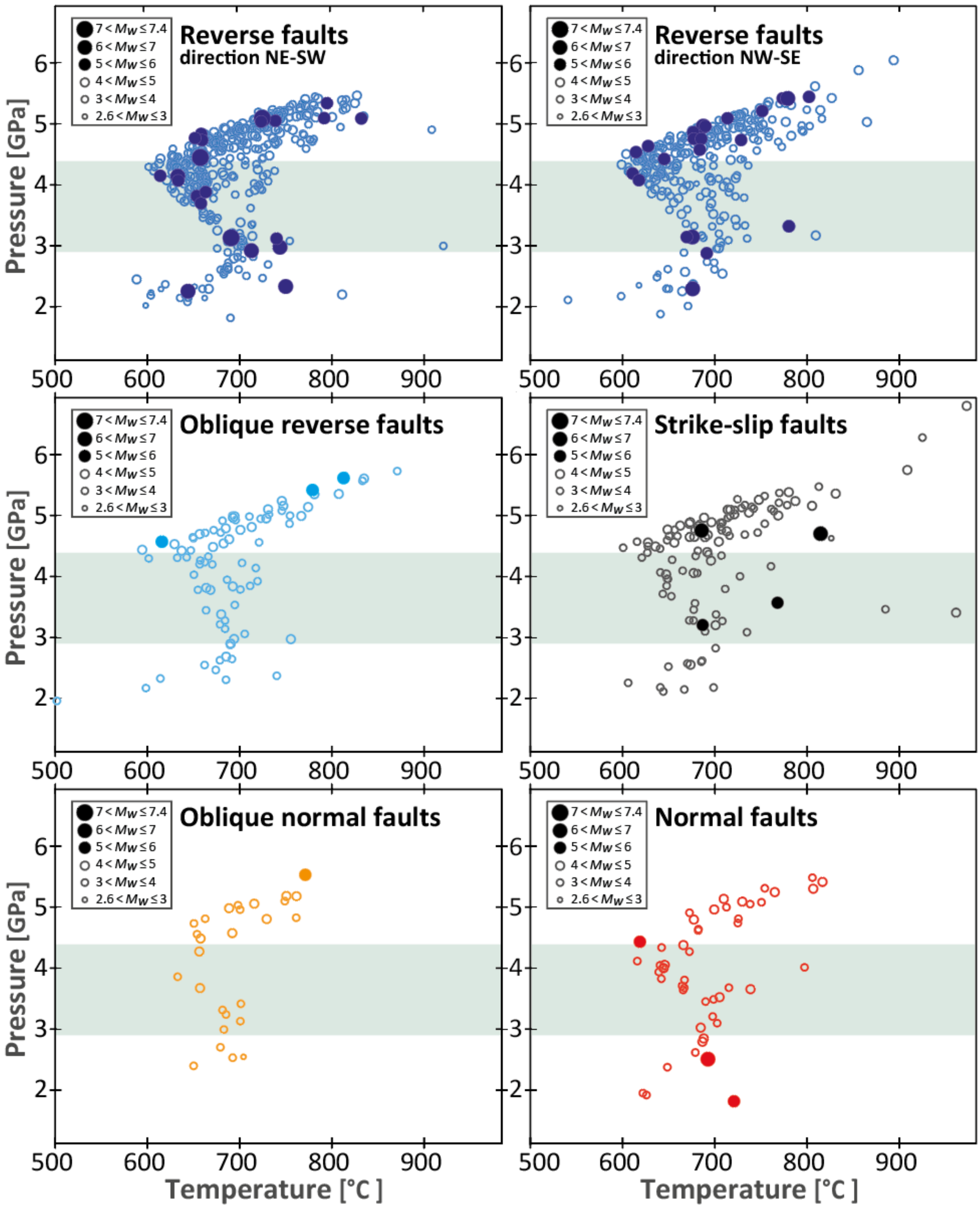

Figure S3 | Extended version of Fig.3a. Detailed view of the depth distribution of focal mechanisms, with an additional comparison between reverse faults that formed normal or parallel to the Vrancea Seismic Body (VSB) and a map to recall the shape of the structure. The green shade accounts for serpentine dehydration with a negative Clapeyron slope.

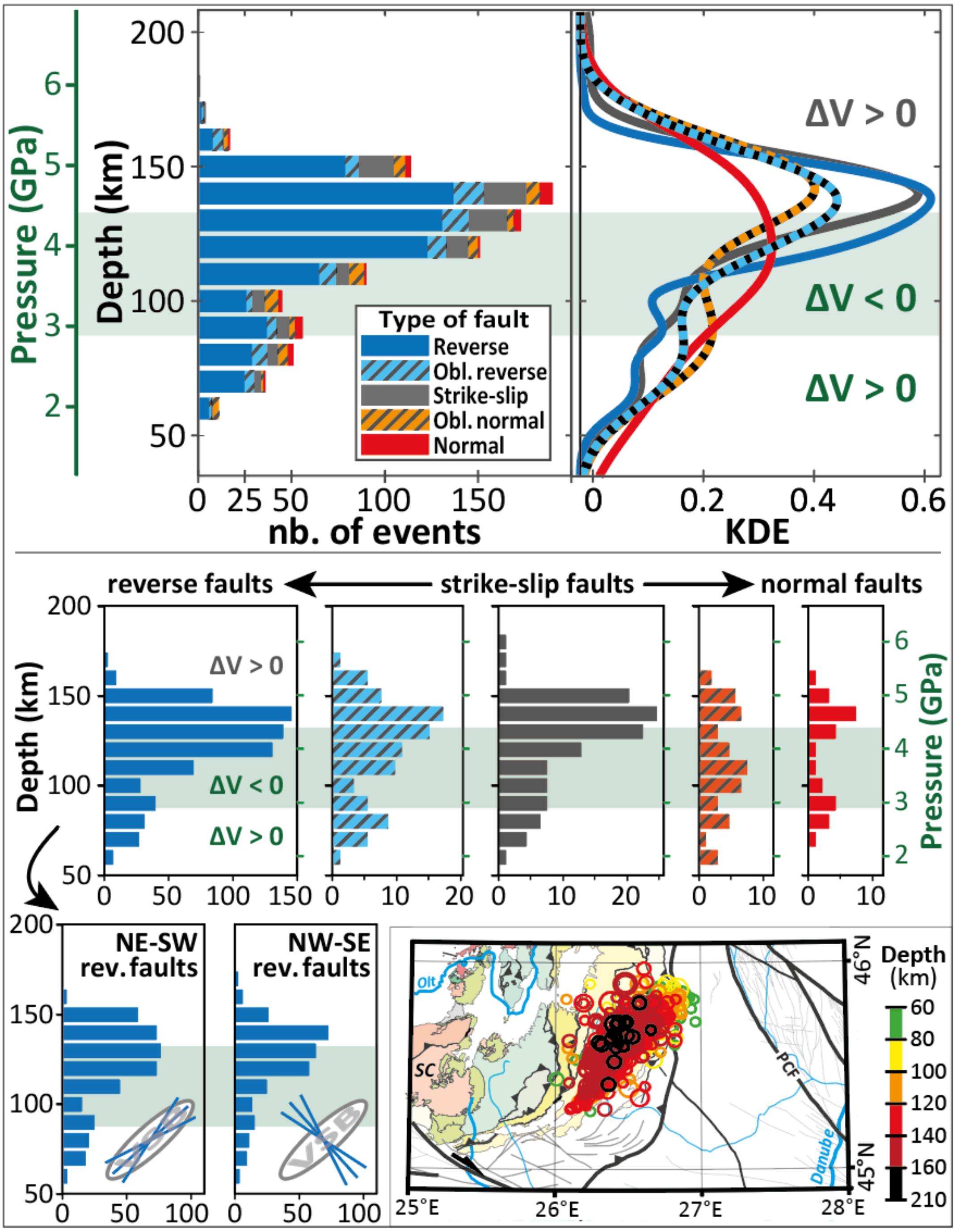

Figure S4 | Extended version of Fig.3b-c. Detailed view of the depth distribution of focal mechanisms, with a comparison between reverse faults with directions normal or parallel to the Vrancea Seismic Body (VSB) and a map to recall the shape of the structure. The green shade accounts for serpentine dehydration with a negative Clapeyron slope. Map modified after Ferrand & Manea (2021). SC: South Caprathians; PCF: Peceneaga-Camena Fault.

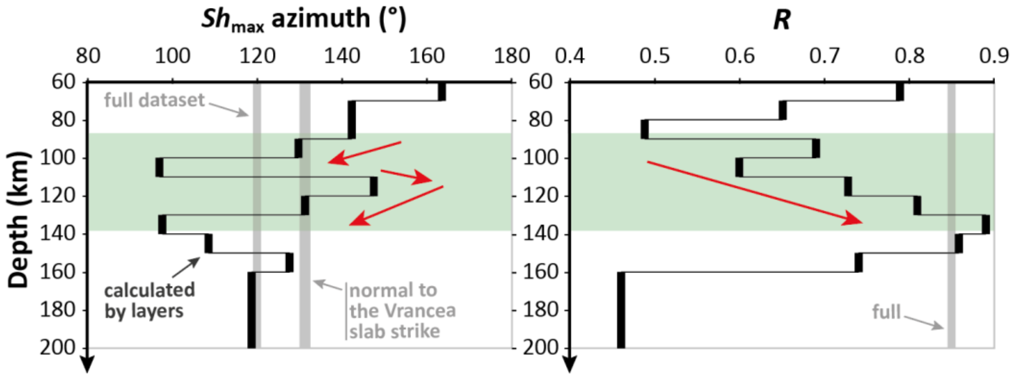

Figure S5 | Depth distribution of the horizontal stress field and associated stress ratio. a) Azimuth of the maximum horizontal compressive stress, compared to the Vrancea slab orientation; **b)** Stress ratio, i.e. relative intensity of σ_2 relative to σ_1 and σ_3, as defined in the *Methods***.** The green shade accounts for serpentine dehydration with a negative Clapeyron slope.

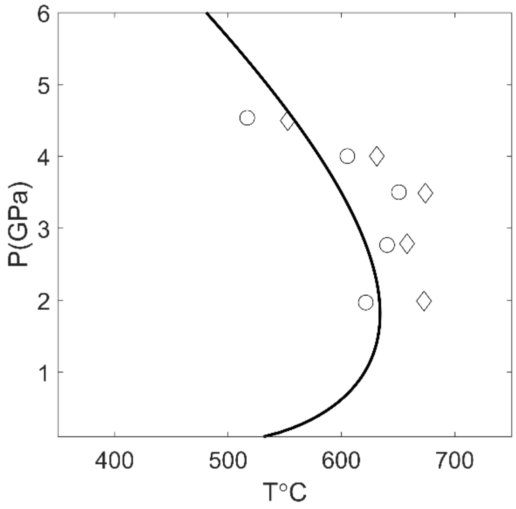

Figure S6 | Antigorite breakdown: model *vs* data. Pressure-temperature diagram showing the antigorite breakdown reaction to form olivine, orthopyroxene, and water in the Mg-Si-O-H system. Symbols indicate the experimentally determined locations of the reaction (Wunder & Schreyer, 1997), with circles and diamond respectively indicating antigorite stability and destabilized.

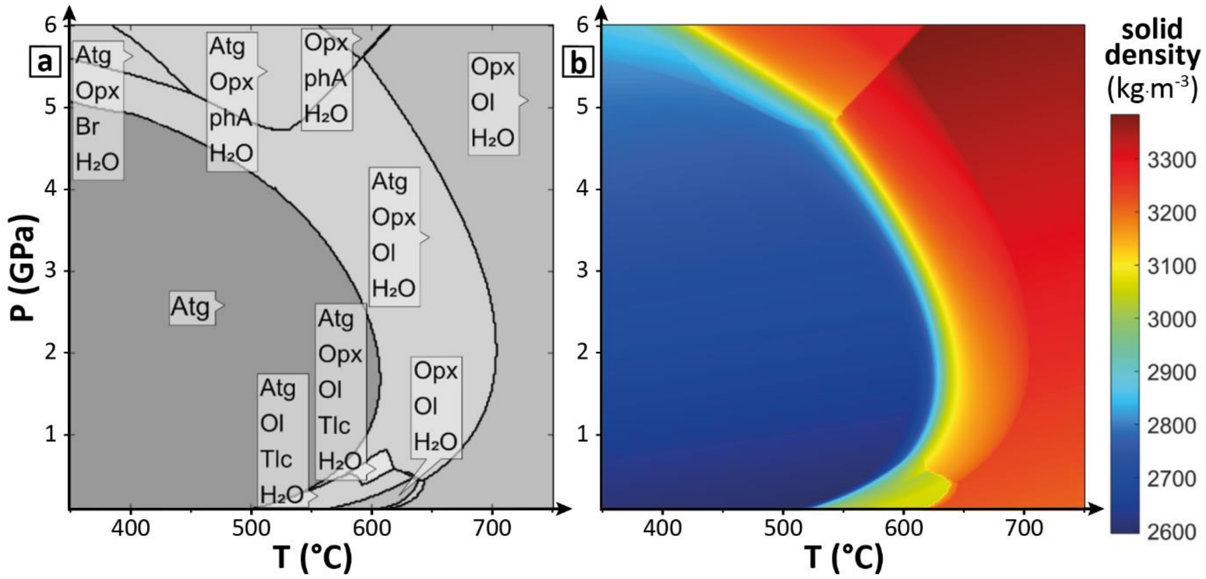

Figure S7 | Phase diagram of an antigorite sample at intermediate depths and associated solid density. a) Phase diagram for a rock consisting of impure antigorite with composition: Mg = 44.5957, Al = 0.6809, Fe = 3.0638, Si = 33.6596, H = 62, O = 147. Minerals: Atg= Antigorite, Opx= Orthopyroxene, Ol = Olivine, Br = Brucite, Tlc = Talc, phA = phase A. Smallest fields are not labelled. Darker gray indicates higher variance. **b)** Density of the solid corresponding to the phase diagram in (a).

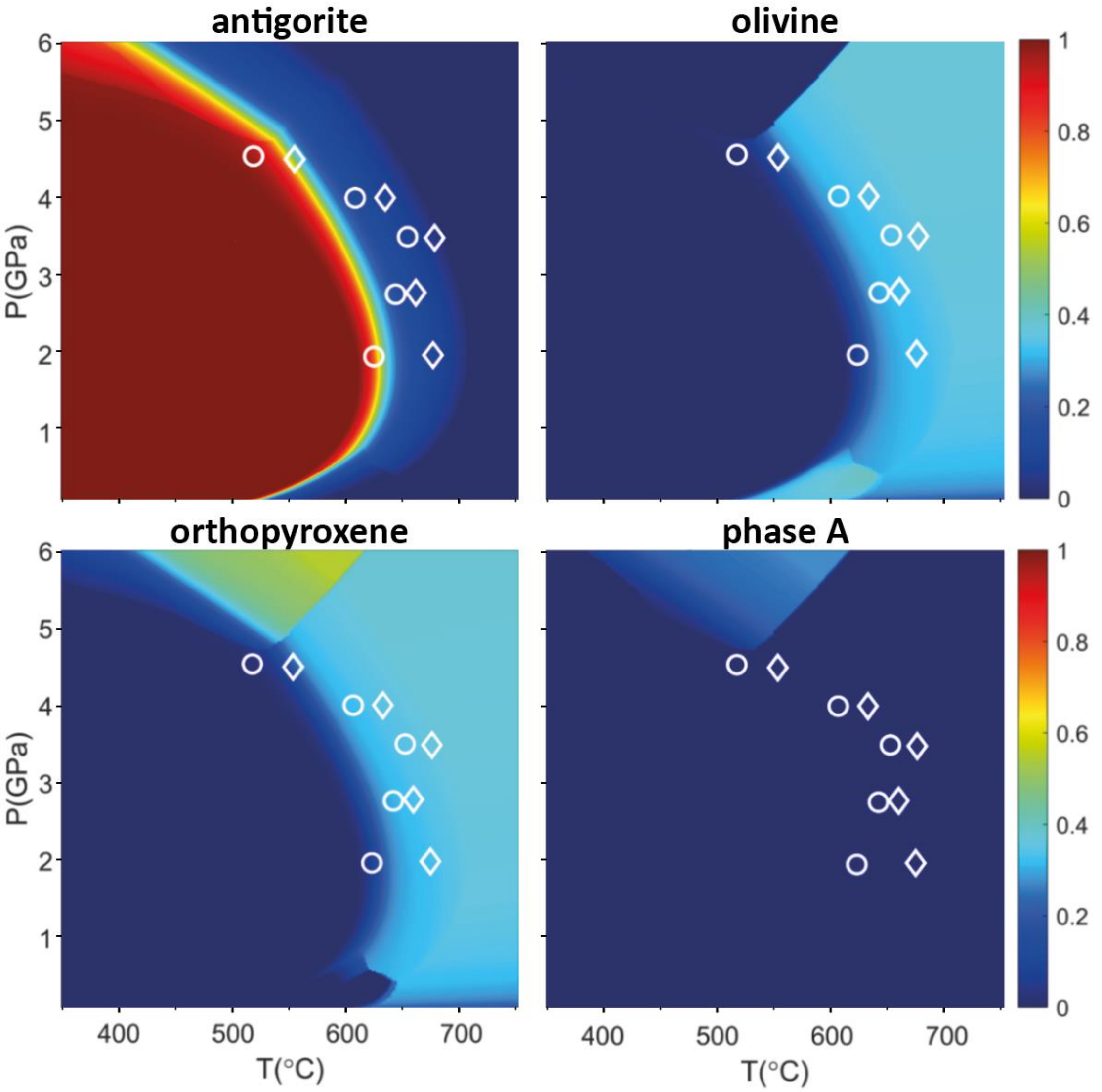

Figure S8 | Abundance of antigorite, olivine, orthopyroxene and phase A. Calculation based on the phase diagram shown in **Fig.S7.** The symbols indicate experimentally determined locations of the Antigorite dehydration from Wunder & Schreyer (1997). The calculated data fit the experimental to a reasonable degree to use the calculations for some first order predictions. Note that in the experiments of Wunder & Schreyer, there was still Antigorite left, and hence the reaction may span a field in P-T space due to the solid-solution behavior of the minerals. Most important here is the curvature of the reaction slope from positive at low pressure to negative at increasing pressure, which is captured also by the calculations.

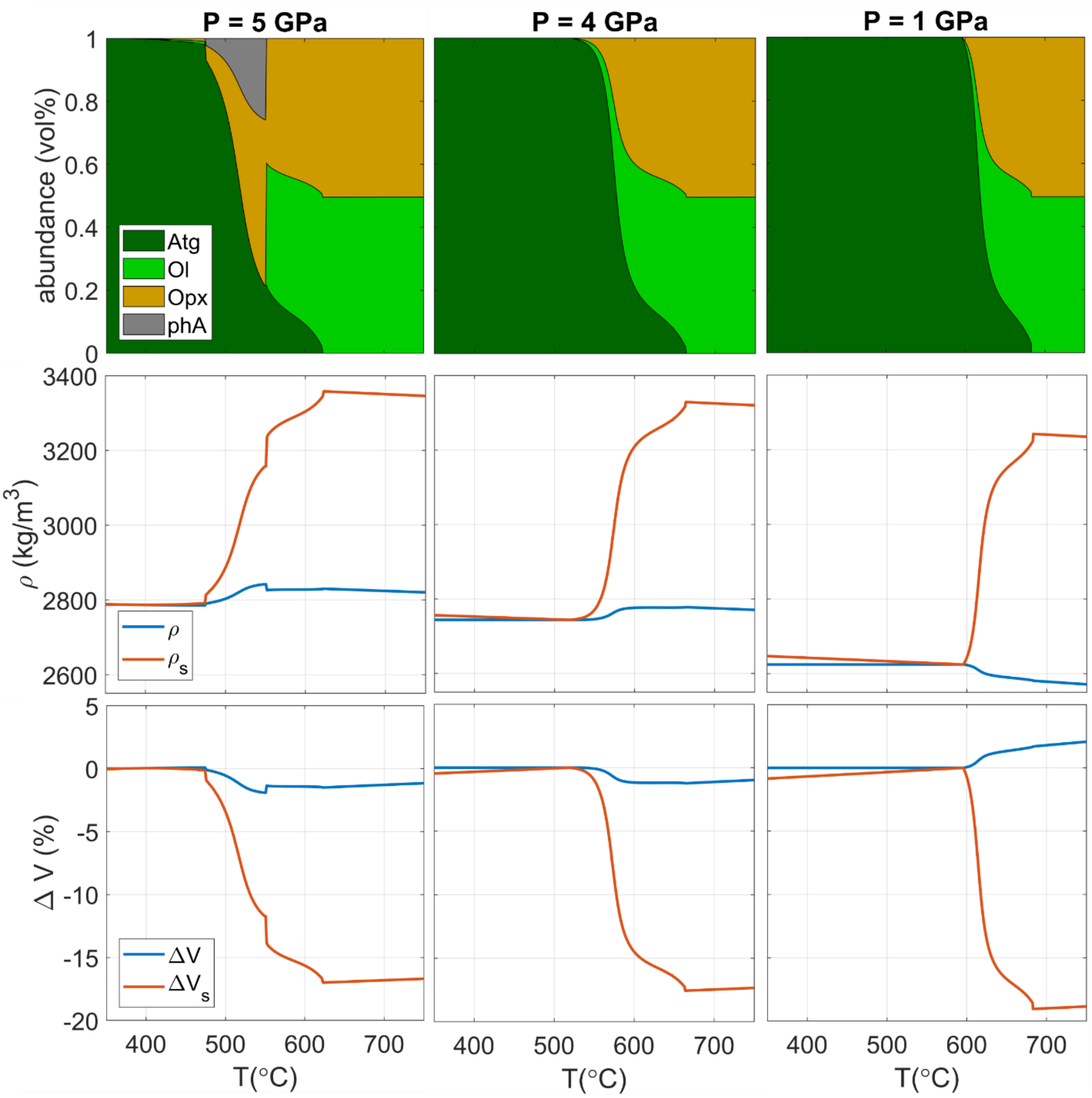

Figure S9 | One-dimensional profiles at three fixed pressures, showing the changes in density of the system, the rock density and the associated volume change as a function of temperature. Where the Clapeyron slope of the reaction is negative, the volume change of the system is also negative. If fluid is allowed to escape, the negative volume change will increase strongly as the remaining dehydrated rock is densified.

References

1. Ferrand, T. P. & Manea, E. F. Dehydration-induced earthquakes identified in a subducted oceanic slab beneath Vrancea, Romania. *Scientific Reports* **11**(1), 1-9 (2021).
2. Frohlich, C. Triangle diagrams ternary graphs to display similarity and diversity of earthquake focal mechanisms. *Physics of the Earth & Planetary Interiors* **75**, 193-198 (1992).
3. Wunder, B. & Schreyer, W. Antigorite: High-pressure stability in the system MgO-SiO_2-H_2O (MSH). *Lithos* **41**(1-3), 213-227 (1997).
